# Inhibition of Hepatitis B Virus (HBV) by Tachyplesin, a Marine Antimicrobial Cell-Penetrating Peptide

**DOI:** 10.3390/pharmaceutics15020672

**Published:** 2023-02-16

**Authors:** Pankhuri Narula, Sankar Kiruthika, Shruti Chowdhari, Perumal Vivekanandan, Archana Chugh

**Affiliations:** Kusuma School of Biological Sciences, Indian Institute of Technology, Hauz Khas, New Delhi 110016, India

**Keywords:** antimicrobial peptide, antiviral peptide, cell-penetrating peptide, hepatitis B virus Tachyplesin (Tpl), virion secretion

## Abstract

We investigate the role of Tachyplesin (Tpl), a marine antimicrobial cell-penetrating peptide, as an anti-HBV agent. Our findings, using confocal microscopy and flow cytometry, demonstrate the internalization of FITC-Tpl in both Huh7 and HepG2 cell lines. Further, our results show that Tpl inhibits the expression of HBV proteins, including hepatitis B surface antigen (HBsAg) and hepatitis B ‘e’ antigen (HBeAg) in cell supernatants of human liver cell lines transfected with 1.3× pHBV. Interestingly Tpl also reduces levels of HBV pre-core RNA and HBV pregenomic RNA, suggesting that Tpl-mediated inhibition occurs at the early stages of HBV replication, including viral transcription. In addition, Tpl led to a significant reduction in levels of hepatitis B virion secretion. In sum, here we demonstrate the potent anti-HBV activity of Tpl at non-cytotoxic concentrations indicating the potential of Tpl to emerge as an effective therapeutic peptide against HBV.

## 1. Introduction

The Hepatitis B virus (HBV) is an enveloped DNA virus with a partially double-stranded relaxed circular DNA (rcDNA) genome of 3.2 kb. HBV can cause chronic infection and is the leading cause of liver cirrhosis and hepatocellular carcinoma (HCC) [1]. Partial double-stranded DNA of HBV consists of a minus(−) strand, which is complimentary to pregenomic RNA (pgRNA), and a shorter plus(+) strand. The minus strand consists of four overlapping open reading frames (ORFs), namely PreC/C, which codes for Hepatitis B ‘e’ Ag (HBeAg) and Hepatitis B core Ag (HBcAg), ORF P, which encodes the HBV DNA polymerase, PreS/S ORF encodes Hepatitis B surface antigen (HBsAg), and ORF X, the smallest ORF, encodes the HBx protein. HBV is hepatotropic [2], and after successful entry into the host hepatocytes and uncoating, the viral genome replicates in the nucleus. In the nucleus, viral rcDNA is repaired and converted into HBV covalently closed circular DNA (cccDNA/viral minichromosome) with the aid of host polymerases and DNA repair proteins. HBV cccDNA acts as a template for transcription for (a) the viral mRNAs, which are translated into viral proteins [3], and (b) the HBV pgRNA, which is packaged into the HBV core protein along with the viral polymerase. The HBV pgRNA serves as the template for reverse transcription, resulting in the generation of HBV DNA. This is followed by the maturation of viral nucleocapsid in the endoplasmic reticulum and viral budding. A significant proportion of HBV core particles with the virus genome may return to the nucleus to maintain HBV cccDNA levels [4].

Currently, interferons (IFNs) and nucleoside analogs (NAs) are used for the management of HBV. NAs act by inhibiting the activity of reverse transcriptase, leading to reduced viral replication. IFNs are known to act through various mechanisms and are found to be effective against a subset of patients. The toxicity and efficacy of antiviral agents, as well as the emergence of antiviral resistance, have been key issues with the existing anti-HBV therapeutic regime [5]. Moreover, since the persistence of cccDNA acts as a reservoir of viral relapse, eradication of HBV cccDNA is the ultimate goal. However, none of the currently approved therapies target HBV cccDNA. 

Antimicrobial peptides (AMPs) are small peptides, also known as host defense peptides (HDPs). They are short amino acid sequences ranging from 5–30 amino acids (aa) and display large structural diversity. In the last few years, AMPs have gained attention due to their diverse range of action as therapeutics. Few AMPs have been identified for their antiviral activity against viruses [6], and multiple mechanisms of action have been elucidated [7]. AMPs are known to block the attachment of the virus to the host cell membrane by interfering with the membrane envelope and viral glycoproteins, prevent fusion of the virus to host cell by interacting with the host cell receptors, and interfere with viral signaling pathways or inhibit enzymes involved in replication and transcription processes such as polymerases and reverse transcriptase [7]. Mucroporin-M1, a scorpion venom-derived antimicrobial peptide, exhibits anti-HBV activity by activating the MAPK pathway, resulting in the inhibition of HBV replication in vitro and in vivo [8]. In addition to AMPs, cell-penetrating peptides (CPPs) such as TAT, Penetratin, and Transportan have been extensively studied for their internalization into mammalian cells [9]. Apart from their ability to translocate themselves across cell membranes, CPPs are known for the efficient delivery of macromolecules [10]. Therefore, CPPs have been used for the delivery of antiviral agents into cells that are not easily permeable. Both AMPs and CPPs exhibit common characteristics such as cationicity and amphiphilicity and, in some instances, have been shown to play interchangeable roles, as observed in the case of Tachyplesin [11]. The antibacterial and antifungal activity of CPPs have been widely reported, but their antiviral role has not been well studied [12].

The antiviral activity of CPPs has been investigated for Herpes Simplex Virus (HSV) and Human Immunodeficiency Virus (HIV). In the case of HBV, CPPs have been primarily studied as delivery vehicles for either peptide nucleic acids (PNAs) or siRNAs [13]. CPP-assisted delivery of siRNA has been reported to show inhibitory effects on HBV DNA, HBV RNA, and other viral proteins in a mouse model of HBV infection [14]. In addition, a recent study has demonstrated the anti-HBV activity of a human telomerase reverse transcriptase-derived peptide [15]. Since HBV resides in the host hepatocytes, the antiviral drug should be able to exhibit cellular translocation without being cytotoxic to the host cell.

The present work aims at evaluating the anti-HBV activity of Tachyplesin (Tpl), an antimicrobial cell-penetrating peptide. Tpl is 19 aa long, stabilized by two disulfide bridges imparting stability. It is derived from hemocytes of horseshoe crab *Tachypleus tridentatus* (K_5_-WCFRVCYRGICYRRCRG-K_15_, Cys3-Cys16, and Cys7-Cys12) [16]. The peptide used in this study is a fragment of the natural peptide, which consists of 77 aa. Tpl is known to exhibit significant cellular uptake in both mammalian as well as plant cell culture systems [11]. Tpl is also known to act as an efficient delivery vehicle for various biomolecules [17]. Further, Tachyplesin has shown inhibition against Gram-positive and Gram-negative bacteria, as well as fungi [18]. The anti-parasitic activity of Tpl against leishmania was reported in our lab recently [19]. Further, it has been shown that Tachyplesin or peptides derived from Tachyplesin exhibit anti-HIV-1 activity by inhibiting HIV-cell fusion. Furthermore, it is also known to obstruct the attachment of viral gp120 to CXCR4 and CCR5 co-receptors [20]. Tachyplesin I was also found to significantly inhibit the infection and replication of Singapore grouper iridovirus (SGIV) and Nervous Necrosis Virus (NNV) by interacting with the viral particles and also by inducing Type-1 interferon response [21].

In this study, the anti-HBV activity of Tpl in a transient HBV cell culture model has been evaluated. The cellular permeability of Tpl has been assessed both qualitatively and quantitatively in Huh7 and HepG2 cells. Further, the cytotoxicity of Tpl in both hepatocyte cell lines has been investigated. The effect of Tpl on the secretion of HBV proteins has also been evaluated. In addition, the ability of Tpl to regulate key HBV transcripts has also been investigated by quantitating HBV pcRNA and HBV pgRNA and hepatitis B virion secretion in Huh7 and HepG2 cells. Overall, the ability of Tpl to act as an anti-HBV agent has been determined by assessing its action at various life cycle stages of HBV at non-cytotoxic concentrations.

## 2. Methods

### 2.1. Peptide Synthesis

Fluorescein isothiocyanate (FITC)-labeled Tachyplesin (Tpl) and non-labeled Tpl were custom synthesized using solid phase peptide synthesis with more than 95% purity from GenPro Biotech, Noida, India. The mass spectroscopy and HPLC data of Tachyplesin are given as supplementary information (Figure S3).

### 2.2. Cell Culture

Human hepatoma cell lines, Huh7 and HepG2, were maintained in Dulbecco’s Modified Eagle’s Medium (DMEM, high glucose 4500 mg/L, L-glutamine, phenol red, without sodium pyruvate and HEPES, Thermo Fischer, Gibco™ 11965092 USA) supplemented with 10% Fetal Bovine Serum (FBS, Thermo Fischer Gibco, Miami, FL, USA) and 1% PenStrep (Thermo Fischer Gibco, USA) and incubated at 37 °C with 5% CO_2_.

### 2.3. HBV Plasmid and Transfection

Huh7 and HepG2 cells were transfected with 1.3× pHBV plasmid using transfection reagent lipofectamine2000 (Invitrogen, Waltham, MA, USA), as described previously [22,23,24]. After 6 h of transfection, the media was replaced by fresh media, and cells were treated with different concentrations of peptide (Tpl). Supernatants were collected, and cells were harvested for RNA extraction after 48 h.

### 2.4. Cellular Uptake Studies

Confocal Laser Scanning Microscopy (CLSM): Huh7 and HepG2 cells were seeded in 24-well plates at a density of 10^5^ cells per well on an autoclaved glass coverslip and incubated at 37 °C and 5% CO_2_. After achieving 80% confluency, media was removed, and cells were washed with 1X phosphate buffer saline (PBS). Thereafter, cells were treated with varying concentrations of FITC-Tpl for 1 h. Following this, media was removed, and cells were treated with 0.05% trypan blue for 10 min to remove any membrane-bound peptide. Cells were then washed twice with PBS and observed under a confocal microscope (Olympus Fluoview FV1200, Olympus Corporation, Tokyo, Japan). The uptake of FITC-labeled mutated Tpl (M-Tpl3) where all arginines are replaced with alanines was also investigated. The uptake of Mut-Tpl3 was also investigated in our previous studies [11,17].

Flow Cytometry: Quantitative assessment of FITC-Tpl internalization into Huh7 and HepG2 cells was carried out using flow cytometry. About 80,000 cells were seeded in 24-well plates and incubated overnight at 37 °C and 5% CO_2._ At 80% confluence, cells were treated with various concentrations of FITC-Tpl for 1 h. After the incubation period, cells were trypsinized using 0.1% trypsin-EDTA solution and washed twice with PBS to remove any unbound peptide. The cell pellet obtained was resuspended in sheath fluid and analyzed on BD FACS Aria III (BD Biosciences, San Jose, CA, USA). A total of 10,000 events were recorded, and data were analyzed using FACS Diva 6.0 software.

### 2.5. Cytotoxicity Analysis

MTT assay was used to identify the non-cytotoxic concentrations of Tpl. Cell viability was measured using MTT (3-(4,5_dimethylthaizol-2-yl)-2,5-diphenyltetrazolium bromide) assay. Approximately 10,000 cells were seeded per well in 96-well plates and kept overnight at 37 °C and 5% CO_2_ to achieve more than 80% confluency. Thereafter, cells were treated with various concentrations of Tpl for 48 h. After 48 h, media was removed, and cells were washed with PBS. MTT reagent was added at a final concentration of 1 mg/mL and kept for 4 h at 37 °C and 5% CO_2_. Thereafter, 100 µL of DMSO was added to dissolve the formazan crystals formed, and absorbance was determined at a wavelength of 570 nm with a background wavelength of 630 nm (Multiskan GO microplate spectrophotometer, Thermo Scientific, Waltham, MA, USA). 

### 2.6. Dual-Luciferase Reporter Assay 

Huh7 and HepG2 cells were seeded at a density of 2×10^4^ in 96-well plates and transfected with psiCHECK-2 dual-luciferase (*Renilla*/firefly) reporter vector (Promega Corporation, Madison, WI, USA) using lipofectamine2000 (Invitrogen, USA) according to the manufacturer’s protocol followed by addition of 12 µM of Tpl. Dual-luciferase assay was performed after 48 h of transfection according to the manufacturer’s instructions, and luminescence was measured using the BioTek Citation 5 plate reader (Agilent, Santa Clara, CA, USA). *Renilla* luciferase (reporter) values were normalized to those of firefly (control) luciferase expression. The fold change in cellular transcription in Tpl-treated cells compared to untreated cells is represented as relative luciferase units (RLU) [24].

### 2.7. Estimation of HBV Proteins by ELISA

Huh7 and HepG2 cells were seeded at a density of 3 × 10^5^ cells per well in 12-well plates and incubated at 37 °C and 5% CO_2_ overnight to achieve >80% confluency. Thereafter, cells were transfected with 1 μg of 1.3× pHBV using transfecting agent lipofectamine 2000, as mentioned above. After 6 h of transfection, media was replaced by fresh media, and cells were treated with various concentrations of Tpl for an incubation period of 48 h. After 48 h, cell supernatants were collected to analyze HBV proteins, and cells were harvested to extract RNA. Commercially available HBsAg (MONOLISA, BioRAD, Hercules, CA, USA) and HBeAg (Diapro, Sesto San Giovanni, Italy) ELISA kits were used for the estimation of HBsAg and HBeAg, respectively, from cell culture supernatants as per given instructions. Dilutions were made so that OD values fell within the linear range, as described previously [25,26].

### 2.8. qRT-PCR Studies 

Total RNA was extracted using TRIzol reagent (Invitrogen, USA) as per the manufacturer’s protocol. The RNA pellet obtained was dissolved in nuclease-free water, and RNA concentration was determined using Nanodrop. Purified RNA was treated with DNase, and cDNA was synthesized from 1 µg of RNA using an iScript cDNA synthesis kit (BioRad, USA). Primers targeting HBV pre-core RNA (pcRNA) and pregenomic RNA (pgRNA) were used for quantification of HBV pcRNA and HBV pgRNA (listed in Table 1), and for quantification of IFN-1 pathway genes, the primers targeted for IFN-α and IFN-β were used. Gene expression was measured using iTaq Universal SYBR Green Supermix (BioRad, USA). GAPDH (Glyceraldehyde 3-phosphate dehydrogenase) was used as a control gene for the normalization of gene levels, respectively.

### 2.9. Estimation of Secreted Virion

Huh7 and HepG2 cells were seeded at a density of 3 × 10^5^ cells per well in 12-well plates and transfected with 1.3× pHBV construct, as described earlier [27,28]. Briefly, 120 µL of supernatant collected after an incubation of 48 h was added to an ELISA microplate coated with anti-HBs antibodies (MONOLISA HBsAg ELISA kit, BioRad, USA) and incubated for 90 min at 37 °C. Thereafter, the microplates were washed with PBS and treated with Proteinase K, and DNA was then extracted using a QIAamp DNA mini kit (Qiagen, Germantown, MD, USA). DNA extracted from the captured virions was quantified with real-time PCR using virion DNA-specific primers.

### 2.10. Primers Used in the Study

The details of primers used in the study are given in Table 1.

**Table 1:** The following primers are used for the qPCR studies [23,29,30].

Primer	Sequence (5′ to 3′)
pgRNA	FP: CACCTCTGCCTAATCATC [23] RP: GGAAAGAAGTCAGAAGGCAA
pcRNA	FP: GGTCTGCGCACCAGCACC [23] RP: GGAAAGAAGTCAGAAGGCAA
Virion	FP: GGTCTGCGCACCAGCACC [23] RP: GAACTTTAGGCCCATATTAGTG
IFN-α	FP: GACTCCATCTTGGCTGTGA [29] RP: TGATTTCTGCTCTGACAACCT
IFN-β	FP: GCTTGGATTCCTACAAAGAAGCA [30] RP: ATAGATGGTCAATGCGGCGTC
GAPDH	FP: TGCACCACCAACTGCTTAGC [23] RP: GGCATGGACTGTGGTCATGAG

### 2.11. Statistical Analysis

The values obtained are from three independent experiments. The results are presented as means ± standard deviation (SD). Differences between control (untreated) and treated groups were determined using Student’s *t*-test. *p*-values < 0.05 are represented by * and ^#^ and are considered statistically significant.

## 3. Results

### 3.1. Internalization of FITC-Tpl in Huh7 and HepG2 Cells

**Figure 1:**
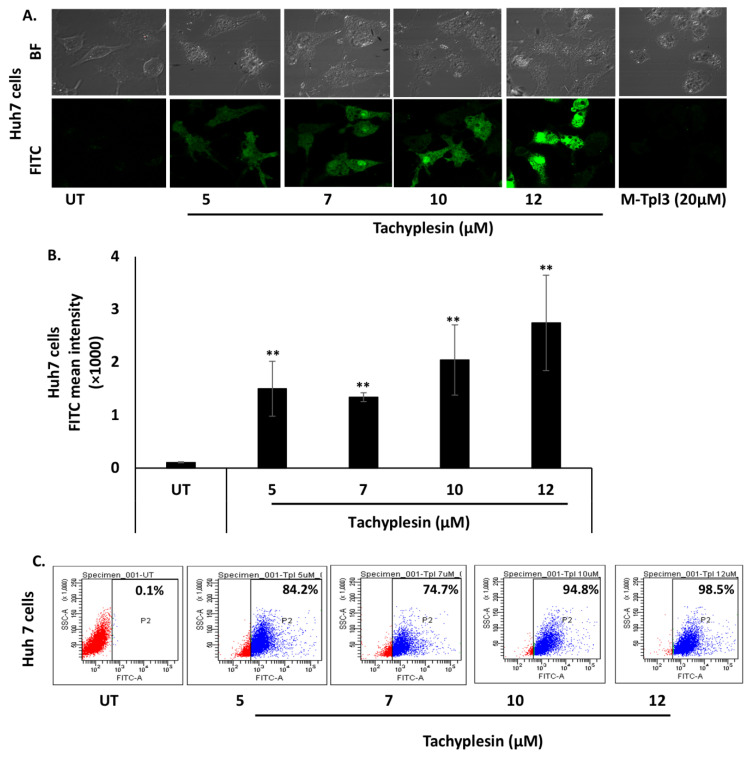

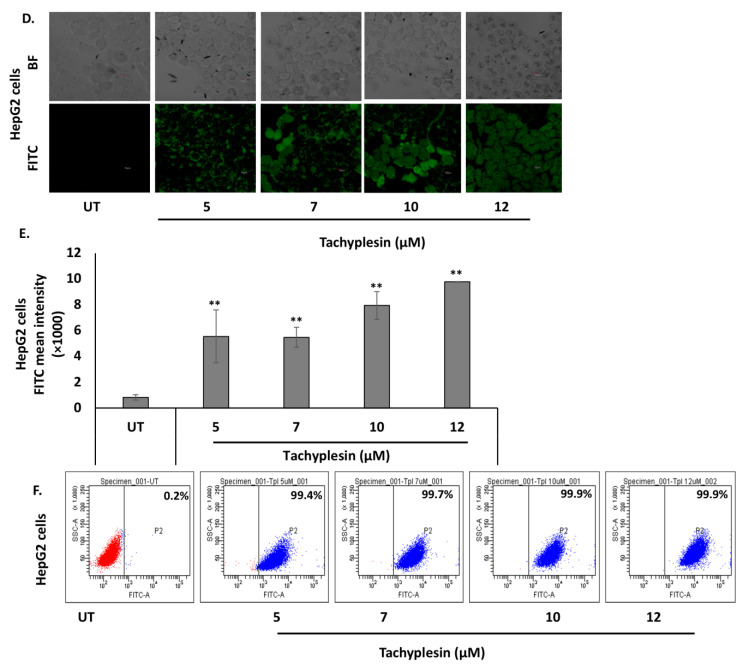
Significant uptake of FITC-Tpl was observed in hepatocyte cell lines at different concentrations of the peptide. (**A**) CLSM studies at magnification of 60× and (**B**,**C**) flow cytometry analysis suggests significant uptake of FITC-Tpl in Huh7 cells. (**D**) CLSM studies at magnification of 40× and (**E**,**F**) flow cytometry analysis show uptake of FITC-Tpl in HepG2 cells. ** represents significant difference with respect to untreated (*p*-value < 0.01, Student’s *t*-test). UT = untreated.

The cell-penetrating ability of Tpl in Huh7 and HepG2 cells was evaluated using confocal microscopy as well as flow cytometry. The data presented in Figure 1A show significant uptake of FITC-Tpl in Huh7 cells at all the concentrations tested (5, 7, 10, and 12 µM). To assess the role of arginine residues in the cellular penetration of Tpl in liver cells, mutated Tpl-3 (M-Tpl3) was employed where all arginines were replaced with alanines. No significant uptake of M-Tpl3 was observed in Huh7 cells as seen by confocal laser scanning microscopy (Figure 1A). Thus, M-Tpl3 was not employed for further antiviral studies.

Further, uptake of FITC-Tpl in Huh7 cells was found to be increasing with an increase in the Tpl concentration, as observed using flow cytometry (Figure 1C). In HepG2 cells, there was appreciable uptake of FITC-Tpl at all the concentrations as observed by confocal microscopy (Figure 1D). Notably, more than 90% of HepG2 cells showed uptake of FITC-Tpl at all concentrations (Figure 1F).

### 3.2. Tpl Is Well Tolerated by Huh7 and HepG2 Cells

**Figure 2:**
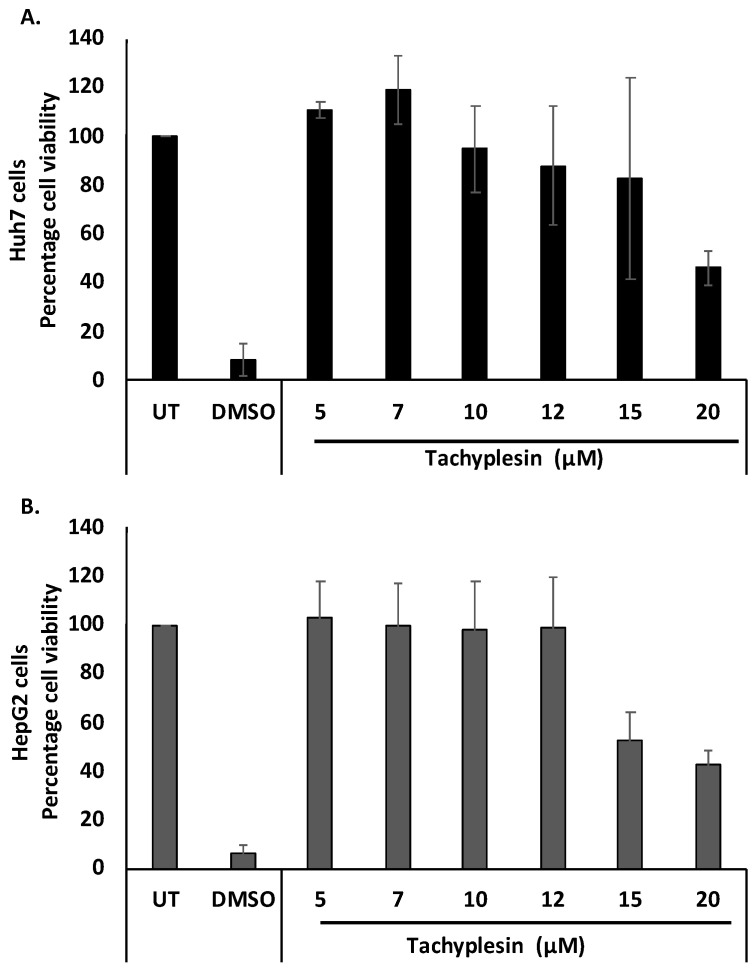
Cytotoxicity studies with Tpl treatment. Cell viability in (**A**) Huh7 cells and (**B**) HepG2 cells after 48 h.

Cellular toxicity associated with Tpl in Huh7 and HepG2 cells was studied using an MTT assay. After 48 h of treatment with up to 12 µM Tpl, cell viability was found to be more than 85% in both cell lines (Figure 2A,B). Therefore, Tpl at 5, 7, 10, and 12 µM concentrations were further used to assess its anti-HBV activity.

### 3.3. Tpl Does Not Affect Transfection or the Cellular Transcription, Translation, and Secretion Machinery

**Figure 3:**
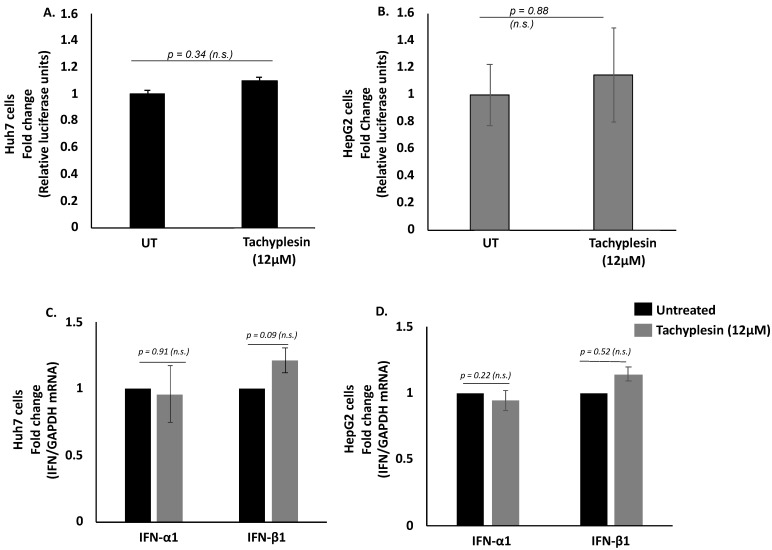
Dual-luciferase assay and IFN-1 expression level. Relative luciferase expression in (**A**) Huh7 and (**B**) HepG2 cells transfected with psiCHECK-2 dual-luciferase (*Renilla*/firefly) reporter vector following 48 h incubation with 12 μM of Tachyplesin. No significant change was observed in levels of IFN-α1 and IFN-β1 (**C**) Huh7 cells and (**D**) HepG2 cells treated with 12 μM of Tpl. *n.s*: non-significant, *p* represents *p*-value.

To ascertain if the Tpl peptide has any effect on other proteins involved in normal cellular transcription or translation machinery, we transfected Huh7 and HepG2 cells with psiCHECK-2 dual-luciferase reporter vector followed by the addition of Tpl peptide. The relative luciferase expression was comparable in cells with or without Tpl, as shown in Figure 3A,B, thus, ruling out inhibition or activation of cellular transcription and translation machinery by Tpl. In addition to the luciferase assay, we also looked at the endogenous levels of two housekeeping genes (GAPDH and β-actin) in both Huh7 and HepG2 cell lines in the presence or absence of Tpl. We found no significant difference in the Ct values of GAPDH and β-actin genes between peptide-treated and untreated cells (Figure S1). In addition, qPCR data for IFNα/β gene expression further confirmed that Tpl (12 µM) treatment did not elicit an interferon response in both Huh7 and HepG2 cells, as shown in Figure 3C,D.

Additionally, to assess the binding affinity of Tpl with HBV DNA at different ratios, a gel retardation assay was performed. With the increase in the ratio of the complex, the intensity of the HBV band decreased, indicating the complexation of HBV DNA with peptide (Figure S2).

### 3.4. Tpl Inhibits Secreted HBV Proteins

The ability of Tpl to inhibit secreted HBV proteins (HBsAg and HBeAg) was estimated using ELISA quantification. HBsAg is secreted by infected hepatocytes, and its levels in the serum have been linked to disease outcome and response to antiviral drugs. Similarly, HBeAg, another secreted HBV protein, is used as an early serum marker of HBV infection, and an HBeAg-positive status has been linked to a high viral load.

**Figure 4:**
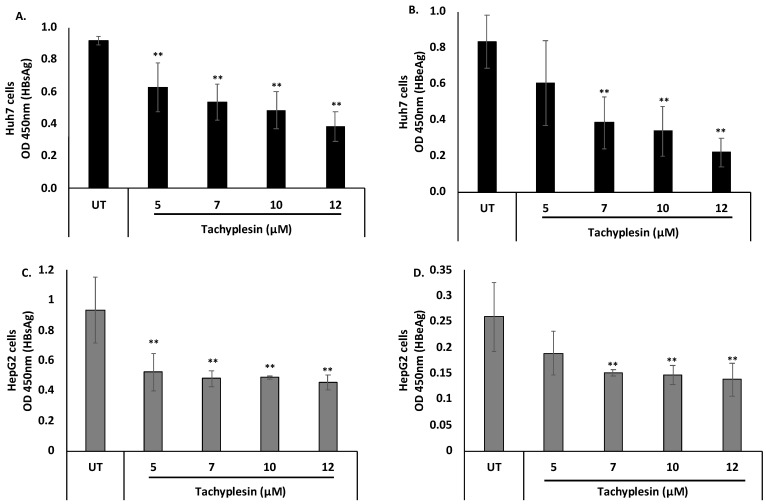
Inhibitory effect of Tpl on the secreted HBV proteins. Huh7 cells and HepG2 cells were transfected with HBV 1.3× plasmid, and supernatants were harvested at 48 h. The inhibitory effect of Tpl on the secretion of (**A**) HBsAg and (**B**) HBeAg was observed in Huh7 cells. (**C**) HBsAg and (**D**) HBeAg levels were also significantly reduced in HepG2 cells treated with Tpl at 7, 10, and 12 µM for 48 h. ** represents significant difference with respect to untreated (*p*-value < 0.01, Student’s *t*-test). UT = untreated.

Notably, treatment with Tpl at 5, 7, 10, and 12 µM in both Huh7 and HepG2 cells resulted in a significant reduction in HBsAg and HBeAg levels as compared to untreated cells (Figure 4). These results suggest that Tpl is a potent inhibitor of both HBsAg and HBeAg in hepatocyte cell lines.

### 3.5. Tpl Inhibits HBV pcRNA and HBV pgRNA

**Figure 5:**
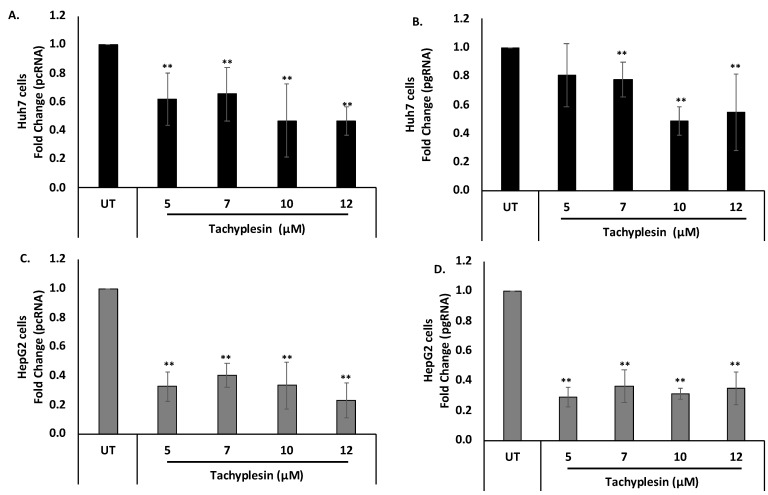
Reduction of HBV pcRNA and HBV pgRNA levels. Tpl led to the reduction of (**A**) HBV pcRNA and (**B**) HBV pgRNA in Huh7 cells. Significant reduction in levels of (**C**) HBV pcRNA and (**D**) HBV pgRNA was also observed in HepG2 cells treated with Tpl. GAPDH was used for normalization. ** represents significant difference with respect to untreated (*p*-value < 0.01, Student’s *t*-test). UT = untreated control.

HBV pgRNA and HBV pcRNA represent widely studied HBV replicative intermediates and transcripts, respectively. Therefore, the effect of Tpl on HBV pcRNA and HBV pgRNA was investigated. HBV pcRNA and HBV pgRNA levels were estimated quantitatively using real-time PCR. Translation of pcRNA leads to the formation of HBeAg. The levels of pcRNA were significantly decreased at all the concentrations of Tpl in both Huh7 (Figure 5A) and HepG2 cells (Figure 5C) as compared to the untreated (UT) control. This finding corroborates with Tpl-mediated reduction in HBeAg, a protein synthesized from HBV pcRNA. Tpl also led to a significant reduction in HBV pgRNA levels in both cell lines as compared to the untreated controls (Figure 5B,D). The HBV pgRNA is packaged into the HBV capsid, where it is converted to HBV DNA by the HBV polymerase. The HBV pgRNA represents a key replicative intermediate that is used as the template for the synthesis of HBV genomic DNA.

### 3.6. Tpl Inhibits Hepatitis B Virion Secretion

**Figure 6:**
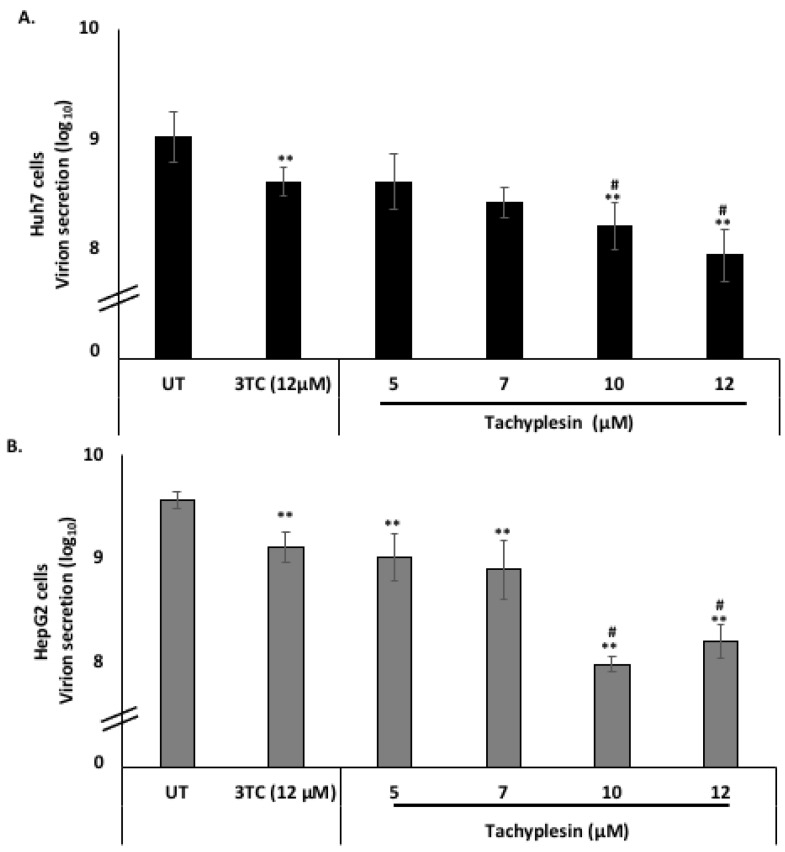
Tpl inhibits hepatitis B virus virion secretion in Huh7 cells and HepG2 cells. Tpl inhibited virion secretion in a concentration-dependent manner in both (**A**) Huh7 and (**B**) HepG2 cell lines. ** represents significant difference with respect to untreated (*p*-value < 0.01, Student’s *t*-test). # represents significant difference with respect to 3TC (*p*-value < 0.05). UT = Untreated, 3TC = lamivudine.

The action of Tpl as an anti-HBV agent was further explored by investigating virion secretion. Virions secreted in the supernatant were quantified using an immunocapture-based protocol followed by quantification of virion-associated DNA using RT-PCR as optimized previously [24,28]. A significant reduction in virion secretion was observed at the concentrations of Tpl employed (Figure 6A,B). The Tpl-mediated reduction in virion secretion was more pronounced than that by lamivudine (3TC) at most of the concentrations tested.

## 4. Discussion

The current treatments available for HBV, such as interferons (IFNs) and nucleoside analogs (NAs), are known to be involved in the suppression of viral replication. However, the side effects, inability to suppress/inhibit cccDNA levels, and development of viral resistance are the major limitations associated with currently available anti-HBV therapies. Therefore, there is a need for alternative therapeutics that can overcome these challenges. Over the last few years, many efforts have been made toward the development of more promising antiviral agents, and the identification of antiviral peptides is one of them [31]. Peptide-based therapeutics have added a new dimension to the area of antiviral research owing to their high efficacy, high selectivity, low cytotoxicity, ease of modification, and small size [7]. Peptides have been known to show antiviral activity by either acting at the virus level, which involves destabilizing viral envelope and damaging virions, or by binding to host cell receptors which are involved in the initial attachment and entry of the virus. A study by Donkers et al. (2019) reported an interaction between Myrcludex B, a synthetic peptide mimicking the sodium taurocholate co-transporting polypeptide (NTCP)-binding domain of HBV and the NTCP-receptor which is a viral entry receptor for the hepatitis B and D virus (HBV/HDV). Myrcludex B was observed to bind non-covalently with the NTCP receptor, blocking HBV and HDV infection [32].

In this study, we identified the anti-HBV activity of antimicrobial cell-penetrating peptide Tpl and its effect on various stages of the HBV life cycle. Previous studies have shown wide spectrum antibacterial, antifungal, and anti-parasitic activities of Tachyplesin, but its efficiency as an anti-HBV agent has not been explored. To achieve anti-HBV activity, the drug molecule should possess efficient cell permeability properties without being cytotoxic to host cells. Taking into consideration these critical factors, such as cell penetration, low cytotoxicity, and antimicrobial activity, Tpl has been investigated in the present study for its antiviral activity against HBV. In both confocal microscopy and flow cytometry analysis, significant uptake of FITC-labeled Tpl was observed in Huh7 cells. However, at all the concentrations of FITC-Tpl, more than 90% of HepG2 cells exhibited fluorescence. Moreover, no significant change was observed in expression levels of luciferase, which suggests that Tpl does not affect cellular transcription or translation.

Current antiviral therapies target viral replication. The loss of serum HBsAg and HBeAg are used as serological indicators of anti-HBV therapy. Thus, the ability of anti-HBV therapies to inhibit the secretion of HBsAg and HBeAg is used for screening antiviral agents [33]. The ability of Tpl to impact the secretion of HBV proteins in both the liver cell lines was evaluated with ELISA. Tpl induced a reduction in HBsAg and HBeAg levels in Huh7 cells. The levels of both HBsAg and HBeAg were significantly reduced in the cell culture supernatants of HepG2 cells treated with Tpl.

Furthermore, we found a significant reduction in levels of HBV pcRNA on treatment with Tpl; this may explain the reduced HBeAg levels observed in the presence of Tpl. The formation of HBV pgRNA from HBV cccDNA in the nucleus is an essential step in the replication of HBV genomic DNA. Tpl was associated with a significant reduction in HBV pgRNA levels. Together, these findings indicate that Tpl may target the early stages of HBV replication, including virus transcription. Finally, Tpl significantly inhibited hepatitis B virion secretion from Huh7 and HepG2 cells. The inhibition of hepatitis B virion secretion in cell culture is an important property of potential anti-HBV agents. Importantly, at all concentrations, Tpl-mediated inhibition of virion secretion was comparable to or more pronounced than that by 3TC (lamivudine).

Overall, our work suggests significant internalization of Tpl into human hepatocyte cell lines, and it also highlights significant anti-HBV activity exhibited by Tpl at non-cytotoxic concentrations. Furthermore, we demonstrate Tpl-mediated inhibition of HBV proteins (HBsAg and HBeAg), HBV transcripts (pcRNA, pgRNA), and virion secretion. It is noteworthy that the ability of Tpl to inhibit hepatitis B virion secretion was comparable to or better than that of lamivudine. The precise mechanism of action of Tpl as an anti-HBV agent needs more elucidation in further studies. Our findings provide promising insights into the anti-HBV activity of Tpl. We believe that this work represents an important step toward the development of peptide-based anti-HBV therapies and their applications.

In sum, this work highlights the anti-HBV activity of Tpl. Importantly, our findings pave the way for further studies on developing efficient peptide-based anti-HBV strategies.

## Data Availability

The data presented in this study are available in this article (and Supplementary Materials).

## Supplementary Materials

The following supporting information can be downloaded at: https://www.mdpi.com/article/10.3390/pharmaceutics15020672/s1, Figure S1: Effect of Tpl on GAPDH and β-actin genes in both the cell lines. The bar graphs represent mean of Ct values from qRT-PCR assays for housekeeping genes: GAPDH and β-actin in Huh7 and HepG2 cell lines treated with or without Tpl. The error bars represent standard deviation (n = 3). Figure S2: Complexation of HBV DNA:Tpl. HBV DNA:Tpl were mixed at different w/w ratios and were then analysed for complexation by gel retardation assay. The band intensity was also quantified using Image lab 6.1 software. Figure S3: MS/HPLC data of Tachyplesin. The expected mass of Tpl was 2449.09 Daltons and the observed mass was found to be 2449.2 (M+3H+ = 817.4) respectively.

## Abbreviations

AMP: antimicrobial peptide; AVP: antiviral peptide; cccDNA: covalently closed circular DNA; CHB: chronic hepatitis B; CLSM: confocal laser scanning microscope; CPP: cell-penetrating peptide; DMEM: Dulbecco’s Modified Eagle’s Medium; DNA: deoxyribonucleic acid; ELISA: enzyme-linked immunosorbent assay; FACS: fluorescence-activated cell sorter; FITC: Fluorescein isothiocyanate; HBV: hepatitis B virus; HBeAg: HBV ‘e’ antigen; HBsAg: HBV surface antigen; IFN: interferon; MTT: (3-(4,5_ dimethylthaizol-2-yl)-2,5-diphenyltetrazolium bromide); NA: nucleotide analog; ORF: open reading frame; PBS: phosphate buffer saline; pcRNA: pre-core RNA; pgRNA: pregenomic RNA; PNA: peptide nucleic acid; rcDNA: relaxed circular DNA; RNA: ribonucleic acid; TAT: transactivator of transcription; Tpl: Tachyplesin; 3TC: Lamivudine.
