# Supplementary material for: Inhibition of Hepatitis B Virus (HBV) by Tachyplesin, a Marine Antimicrobial Cell-Penetrating Peptide
Source: Pharmaceutics. 2023 Feb 16;15(2):672. doi: 10.3390/pharmaceutics15020672 (PMC9962029; doi:10.3390/pharmaceutics15020672)
Supplement: Supplementary file 1 [file pharmaceutics-15-00672-s001.zip › pharmaceutics-1963794-supplementary.pdf]

Supplementary Information

Inhibition of Hepatitis B Virus (HBV) by Tachyplesin, a Marine Antimicrobial Cell-Penetrating Peptide

Pankhuri Narula, Sankar Kiruthika, Shruti Chowdhari, Perumal Vivekanandan * and Archana Chugh *

Kusuma School of Biological Sciences, Indian Institute of Technology, Hauz Khas, New Delhi 110016, India;
* Correspondence: Dr. Archana Chugh (achugh@bioschool.iitd.ac.in) and
Dr. Vivekanandan Perumal (vperumal@bioschool.iitd.ac.in)

Figure S1

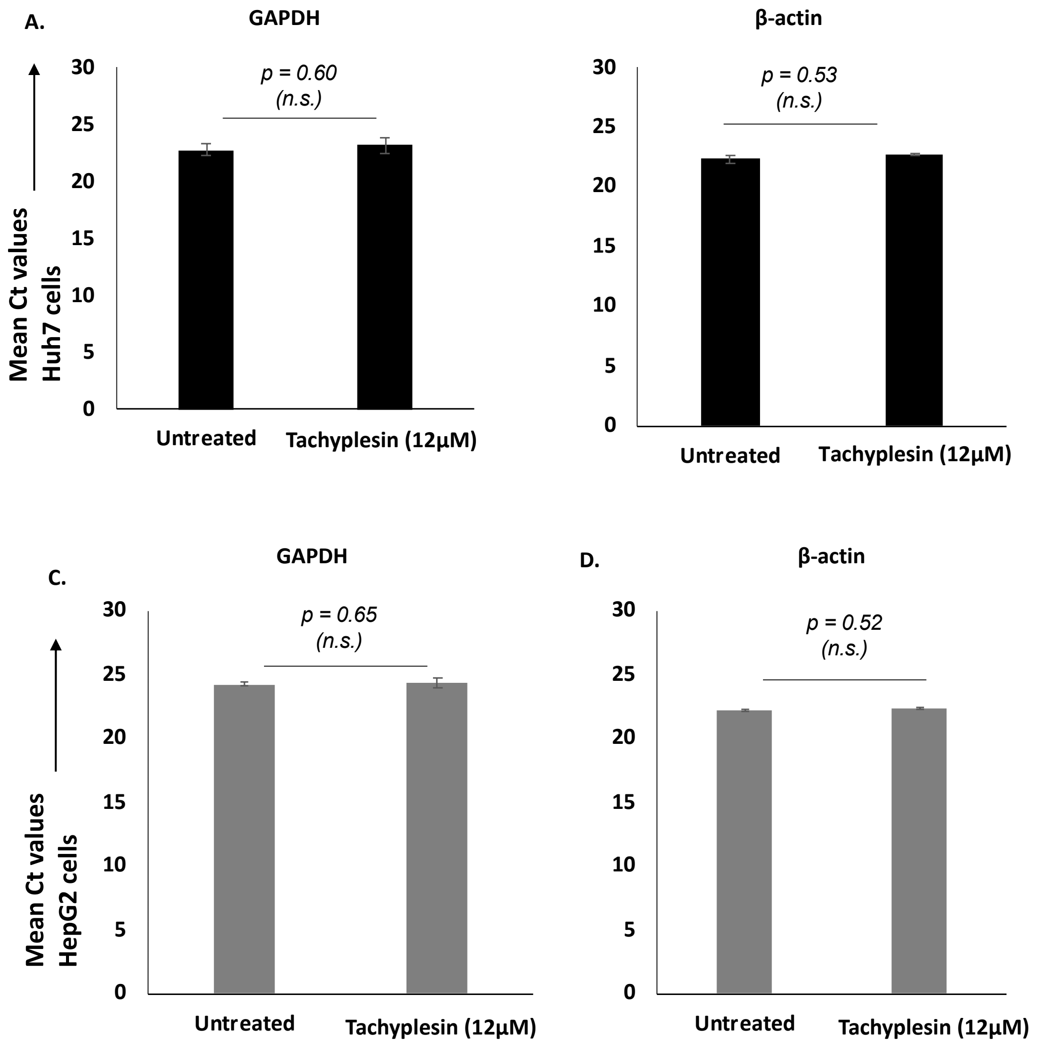

Figure S1: Effect of Tpl on GAPDH and β-actin genes in both the cell lines. The bar graphs represent mean of Ct values from qRT-PCR assays for housekeeping genes: GAPDH and β-actin in Huh7 and HepG2 cell lines treated with or without Tpl. The error bars represent standard deviation (n=3).

Figure S2

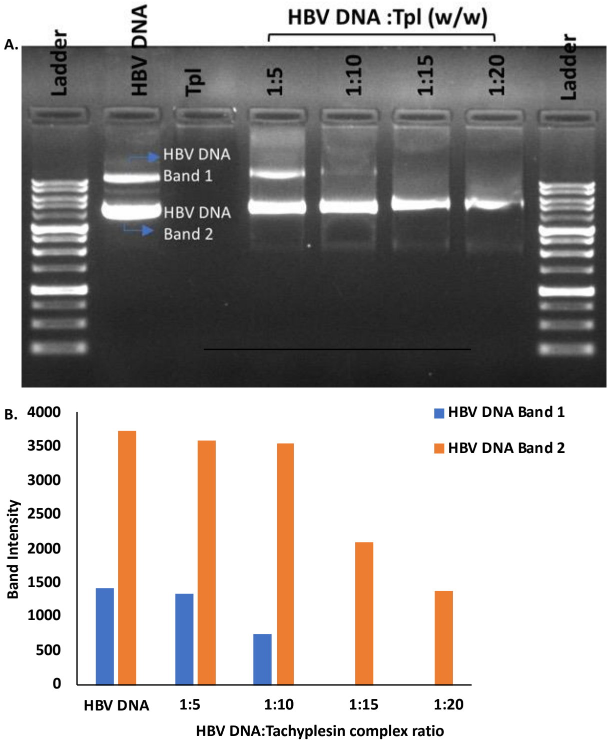

Figure S2: Complexation of HBV DNA:Tpl. HBV DNA:Tpl were mixed at different w/w ratios and were then analysed for complexation by gel retardation assay. The band intensity was also quantified using Image lab 6.1 software.

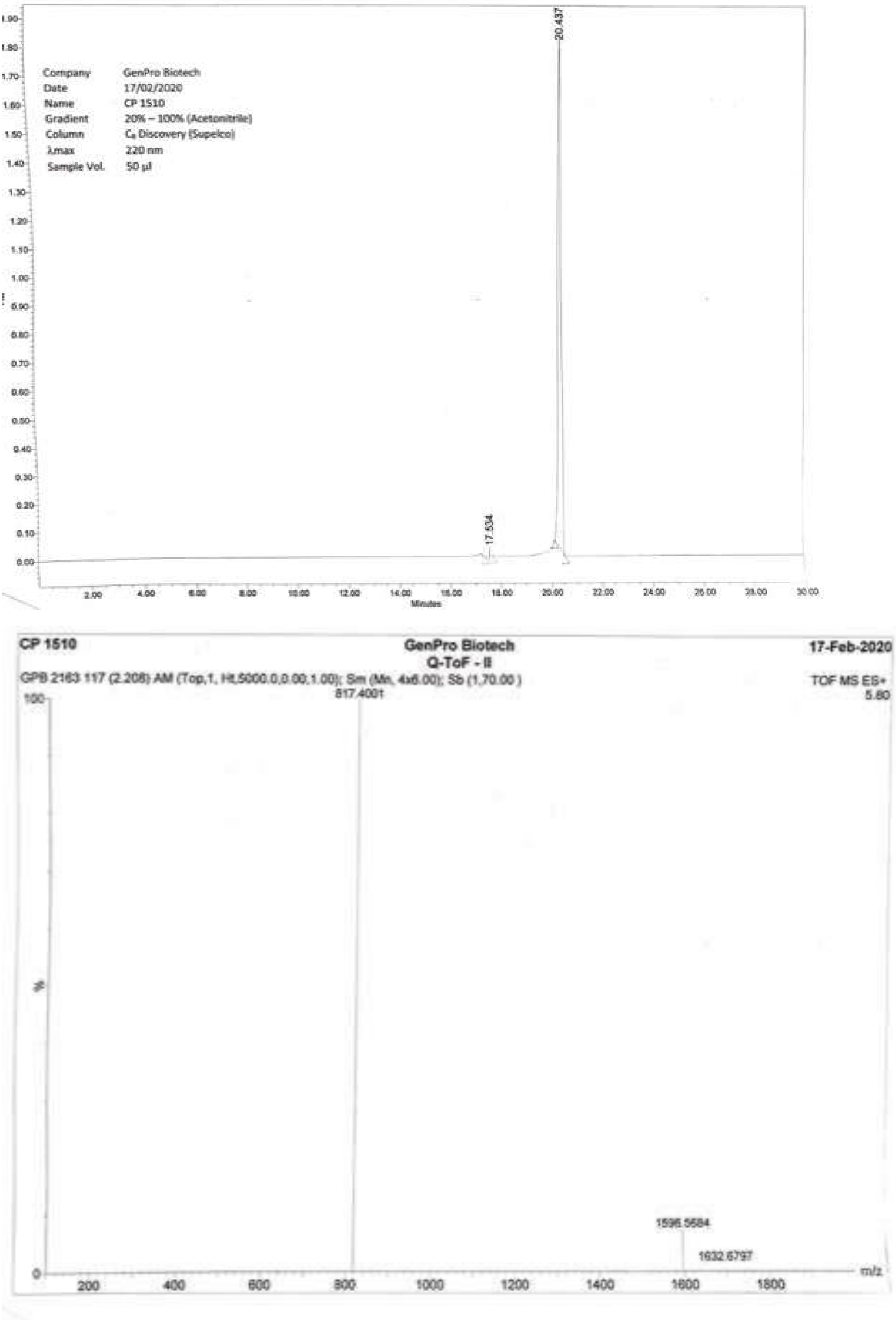

Figure S3: MS/HPLC data of Tachyplesin. The expected mass of Tpl was 2449.09 Daltons and the observed mass was found to be 2449.2 ($M+3H^+ = 817.4$) respectively.
